# Diagnostic value of miR-145 and its regulatory role in macrophage immune response in tuberculosis

**DOI:** 10.1590/1678-4685-GMB-2019-0238

**Published:** 2020-05-29

**Authors:** Yinghui Fu, Xue Yang, Hongyan Chen, Yugang Lu

**Affiliations:** 1 The Fourth Department of Tuberculosis, Shenyang Chest Hospital, Shenyang, Liaoning 110034, China.; 2 Department of Science and education, Shenyang Chest Hospital, Shenyang, Liaoning 110034, China.; 3 Tongji University School of Medicine, Department of Anesthesiology, Shanghai Pulmonary Hospital, Shanghai 200433, China.

**Keywords:** Tuberculosis, microRNA-145, diagnosis, immune response, proliferation

## Abstract

Tuberculosis (TB) induced by *Mycobacterium tuberculosis* (Mtb) is a serious global health burden. This study sought to investigate the expression and diagnostic value of serum miR-145 in TB patients and explore the biological function of miR-145 using macrophages. Serum expression levels of miR-145 were estimated by quantitative real-time PCR. A receiver operating characteristic curve was plotted to evaluate the diagnostic accuracy of miR-145. This study further focused on the effects of miR-145 on cell viability and inflammation in macrophages upon Mtb infection, and explored the potential target gene of miR-145. Serum expression levels of miR-145 were decreased in TB patients, and the upregulated inflammatory cytokines in TB patients were negatively correlated with the serum expression levels of miR-145. miR-145 had considerable diagnostic accuracy in distinguishing of TB patients from healthy individuals and differentiating between active TB cases and latent TB cases. Mtb infection induced an increase in cell viability and inflammatory responses in macrophages, but these promoting effects were rescued by the overexpression of miR-145. CXCL16 was determined as a target gene of miR-145 in macrophages. Overall, this study demonstrated that the decreased serum miR-145 expression serves a candidate diagnostic biomarker in TB patients. The overexpression of miR-145 in macrophages upon Mtb infection can suppress cell viability and infection-induced inflammation via regulating CXCL16, indicating the potential of miR-145 as a therapeutic target of TB.

## Introduction

Tuberculosis (TB) occurrs mainly due to infection by Mycobacterium tuberculosis (Mtb) and remains one of the serious health burdens worldwide, with approximately 10 million new cases detected and 1.5 million deaths every year (Zumla *et al.*, 2015; Evans *et al.*, 2016). Most of TB cases are latent without any typical clinical manifestation, and approximately 10% of these cases can progress to active TB, which has a high mortality rate (Lui *et al.*, 2014). Early diagnosis is still the major challenge for the treatment of TB due to the increasing morbidity and mortality caused by diagnostic delays and misdiagnoses (Chao *et al.*, 2016). Currently, the screening of TB patients mainly relies on smear microscopy, solid culture, chest radiography and tuberculin skin tests (Wallis *et al.*, 2010). However, the feasibility of these established diagnostic methods may vary by the sampling and disease status of TB patients. Bacterial culture is considered the gold standard for TB diagnosis, but it requires a long time to obtain the examination results (Reed *et al.*, 2017). Thus, efforts should be made to develop more effective diagnostic methods.

Mtb infection activates the host immune defense involving innate immune cells, including macrophages, natural killer cells and dendritic cells, and leading to the increased secretion of various inflammatory cytokines, such as interleukin (IL)-1β and tumor necrosis factor (TNF)-α (Andreu *et al.*, 2017). Macrophages as the first barrier against Mtb infection (Liu *et al.*, 2017). Therefore, understanding the mechanisms underlying the Mtb-macrophage interaction may provide novel therapeutic strategies for TB.

Aberrant expression of microRNAs (miRNAs) has been detected in various human diseases, which has attracted increasing attention due to their important clinical significance and biological function (Tantawy *et al.*, 2018; Chen *et al.*, 2019; Zhang *et al.*, 2019). miRNAs are a group of small noncoding RNAs with regulatory effects on gene expression at the posttranscriptional level (Diao *et al.*, 2018). Various cellular processes, such as proliferation, migration, invasion and apoptosis, can be modulated by the functional miRNAs (Wang *et al.*, 2019). For example, the overexpression of miR-182 and miR-183 could facilitate mesothelioma cell proliferation, migration and adhesion abilities, indicating their potential as therapeutic molecules (Suzuki *et al.*, 2018). Downregulation of microRNA-145 (miR-145) expression has been found in macrophages after the infection with Mtb (Furci *et al.*, 2013), but its precise role in TB remains unclear.

This study was performed with the aim of investigating the expression patterns and diagnostic potential of miR-145 in serum samples of TB patients and further assessing the effects of miR-145 on cell viability and the inflammatory response of macrophages upon Mtb infection. The data of this study demonstrated that the decreased expression of miR-145 had relatively high diagnostic accuracy in distinguishing TB patients from healthy individuals, and that the viability and inflammation in macrophages upon Mtb infection could be inhibited by miR-145. This study provides evidence for miR-145 to serve as a candidate diagnostic biomarker and a promising therapeutic target for TB treatment.

## Material and Methods

### Patients and serum collection

A total of 150 subjects, including healthy controls (HC, n = 50), latent TB patients (LTB, n = 40) and active TB patients (ATB, n = 60), were recruited in this study from Shenyang Thoracic Hospital between 2014 and 2017. The approval from the Ethics Committee of Shenyang Thoracic Hospital was obtained for this study, and informed consent was signed by each participant. ATB was confirmed by the clinical symptoms, microscopy and bacterial culture. The patients without typical clinical symptoms but with positive quantiFERON test were determined as LTB group. Serum was isolated from the blood samples that collected from the patients and healthy controls and stored at −80°C for further analyses.

### Cell culture and Mtb infection

Human macrophage THP-1 and murine macrophage RAW264.7 were purchased from the Cell Bank in Shanghai Institutes for Biological Sciences (Shanghai, China) and cultured in RPMI-1640 medium (Gibco, CA, USA) added with 10% fetal bovine serum (FBS; Gibco, CA, USA) in a humidified atmosphere with 5% CO_2_ at 37°C.

Mtb strain H37Rv was obtained from ATCC and grown in Middlebrook 7H9 broth medium supplemented with 10% acid albumin dextrose catalase enrichment (OADC; Sigma, St. Louis, MO). Cells were infected at a multiplicity of infection of 10 bacteria per cell (MOI 10) for 6 h. The cells were washed using RPMI1640 medium for 6 times to remove the rest bacteria.

### Cell transfection

To regulate the expression of miR-145 in the macrophages, miR-145 mimic, miR-145 inhibitor and their corresponding negative controls (mimic NC and inhibitor NC) were synthesized in GenePharma (Shanghai, China). These vectors were separately transfected into the macrophages using Lipofectamine 3000 (Invitrogen, Carlsbad, CA, USA) following the manufacture's instruction. After 48 h of transfection, the cells were used for further experiments.

### RNA extraction and quantitative real-time PCR (qRT-PCR)

Total RNA was extracted from serum and cells by TRIzol reagent (Invitrogen, Carlsbad, CA, USA) in accordance with the manufacture's instruction. Single-stranded cDNA was obtained from the RNA by the commercial Revert Aid First Strand cDNA Synthesis Kit (Thermo Fisher Scientific, USA). miR-145 expression was estimated using qPCR, which was carried out using SYBR green I Master Mix kit (Invitrogen, Carlsbad, CA, USA) on a 7500 Real-Time PCR System (Applied Biosystems, USA). U6 was used as an internal control for miR-145. The final relative expression was calculated using the 2^-Ä^ΔCt method.

### Enzyme-linked immunosorbent assay (ELISA)

Concentration of the inflammatory cytokines, including IL-1β and TNF-α, in the serum samples and supernatant of cell cultures were detected using ELISA kit (Thermo Fisher Scientific, USA) according to the manufacturer's protocol.

### Cell viability assay

Viability of macrophages was investigated using Cell Counting kit-8 (CCK-8) assay (Beyotime, Shanghai, China). The cells with a density of 3000 cells/well was seeded into 96-well plates and cultured at 37°C for 72 h. The CCK-8 reagent was added into the cells every 24 h with further 4 h incubation. The absorbance at 450 nm was measured using a microplate reader (Molecular Devises, CA, USA).

### Luciferase reporter assay

According to bioinformatics analysis with miRWalk (http://zmf.umm.uni-heidelberg.de/apps/zmf/mirwalk/micrornapredictedtarget.html), we predicted a potential target gene chemokine (C-X-C motif) ligand 16 (CXCL16) for miR-145. To verify the interaction between miR-145 and CXCL16 in macrophages, a l3uciferase reporter assay was performed. The wild type 3’-UTR of CXCL16 (WT) and mutant type 3’-UTR of CXCL16 (MT) were synthesized and separately cloned into luciferase reporter vector. The combined vectors were co-transfected into the macrophages with miR-145 mimic, miR-145 inhibitor or the NCs using Lipofectamine 3000 (Invitrogen, Carlsbad, CA, USA). Luciferase activity was evaluated by a Dual-Luciferase Reporter Assay System (Promega).

### Statistical analysis

All data were expressed as mean ± SD and analyzed using SPSS 18.0 software (SPSS Inc., Chicago, IL) and GraphPad Prism 5.0 software (GraphPad Software, Inc., USA). Differences between groups were assessed using Student's t-test and one-way ANOVA. Correlations between parameters were analyzed by Pearson correlation coefficient. Receiver operating characteristics curve (ROC) was plotted to evaluate the diagnostic performance of miR-145 in TB patients. A *P-value* of less than 0.05 indicates statistically significant.

## Results

### Baseline characteristics of the study cohort

The demographic and clinical characteristics of the study cohort were summarized in Table 1. There was no difference in age or gender between the HC, LTB and ATB groups (both *P* > 0.05). The serum concentrations of IL-1β and TNF-α were different between the three groups (all *P* < 0.001), and the higher levels of the two inflammatory cytokines were observed in the TB patients than in the healthy individuals.

**Table 1:** Demographic and clinical characteristics of the study population

Features	Groups			*P-value*
	HC (n = 50)	LTB (n = 40)	ATB (n = 60)	
Age	33.2 ± 6.91	31.95 ± 8.14	32.07 ± 8.07	0.667
(years; mean ± SD)
Gender	29/21	22/18	38/22	0.689
(male/female)
IL-1β	14.64 ± 4.58	51.58 ± 10.01	101.23 ± 16.03	< 0.001
(pg/mL)
TNF-α	3.79 ± 1.28	10.56 ± 2.65	36.67 ± 9.46	< 0.001
(pg/mL)

HC, healthy control; LTB, latent tuberculosis; ATB, active tuberculosis; IL, interleukin; TNF, tumor necrosis factor.

### Serum expression levels of miR-145 in the patients with TB

**Figure 1:**
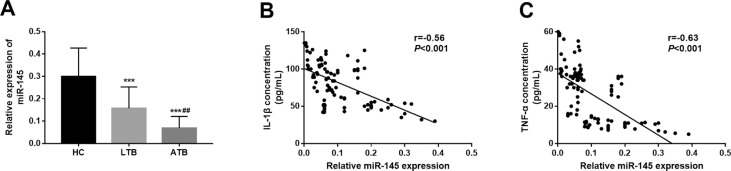
Serum expression of miR-145 and its correlation with serum levels of inflammatory cytokines. A. Serum expression of miR-145 was decreased in the TB patients compared with the healthy controls, and the lowest expression of miR-145 was observed in the ATB patients. B and C. Serum expression of miR-145 was negatively correlated with the concentrations of IL-1β (B; r = −0.56, *P* < 0.001) and TNF-α (C; r = −0.63, *P* < 0.001). HC, healthy control; LTB, latent tuberculosis; ATB, active tuberculosis; **P < 0.01, ***P < 0.001 vs. HC; ^##^ *P* < 0.01 vs. LTB.

The expression of miR-145 levels were measured in the serum samples collected from the research participants. As shown in Figure 1A, the expression of miR-145 was significantly decreased in LTB and ATB patients when compared to the HC group (both *P* < 0.001). Compared with that in the LTB group, we observed a marked reduction in the miR-145 expression in the patients with ATB (*P* < 0.01).

### miR-145 levels and inflammatory cytokines correlation in patients with TB

Considering the critical role of inflammatory reactions in the development of TB, the relationship between miR-145 expression and inflammatory cytokines in TB patients was examined in this study. From Figure 1B and 1C, we found that the serum expression levels of miR-145 were negatively correlated with the serum concentrations of IL-1β (r = −0.56, *P* < 0.001) and TNF-α (r = −0.63, *P* < 0.001) in the TB patients.

### Diagnostic accuracy of miR-145 in TB patients

**Figure 2:**
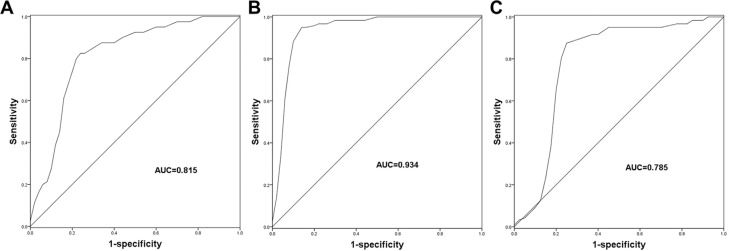
ROC curves based on the serum expression of miR-145. A. miR-145 could be used to distinguish LTB patients from the healthy controls with an AUC of 0.815. B. miR-145 had high diagnostic value for the differentiation between ATB patients and healthy controls with an AUC of 0.934. C. miR-145 had the diagnostic potential to screen ATB cases from the LTB cases with an AUC value of 0.785. HC, healthy control; LTB, latent tuberculosis; ATB, active tuberculosis; AUC, area under the ROC curve.

Given the deregulated serum miR-145 expression in TB patients, this study further investigated the diagnostic value of miR-145. For the patients with LTB, miR-145 was demonstrated to have considerable diagnostic accuracy in distinguishing LTB cases from healthy controls with an area under the curve (AUC) of 0.815 (cutoff value = 0.235, sensitivity = 82.5%, specificity = 76.0%; Figure 2A and Table 2). As expected, a ROC curve for the ATB patients (Figure 2B) also showed a relatively high diagnostic accuracy of miR-145 for the differentiation between ATB patients and healthy controls (AUC = 0.934, cutoff value = 0.155, sensitivity =95.0%, specificity = 86.0%; Table 2). Furthermore, we also explored the diagnostic performance of miR-145 for differentiating ATB patients from the LTB cases. As shown in Figure 2C, a ROC curve with an AUC value of 0.785 was obtained with a sensitivity and specificity of 88.3% and 75.0%, respectively, at a cutoff value of 0.098 (Table 2), indicating a moderate diagnostic value of miR-145.

**Table 2:** Diagnostic accuracy, sensitivity and specificity of miR-145 for TB patients

Groups	AUC	Cutoff value	Sensitivity	Specificity
LTB *vs.* HC	0.815	0.235	82.5%	76.0%
ATB *vs.* HC	0.934	0.155	95.0%	86.0%
ATB *vs.* LTB	0.785	0.098	88.3%	75.0%

TB, tuberculosis; HC, healthy control; LTB, latent tuberculosis; ATB, active tuberculosis; AUC, area under the curve.

### Changes in cell viability, inflammation and miR-145 expression in macrophages after Mtb infection

**Figure 3:**
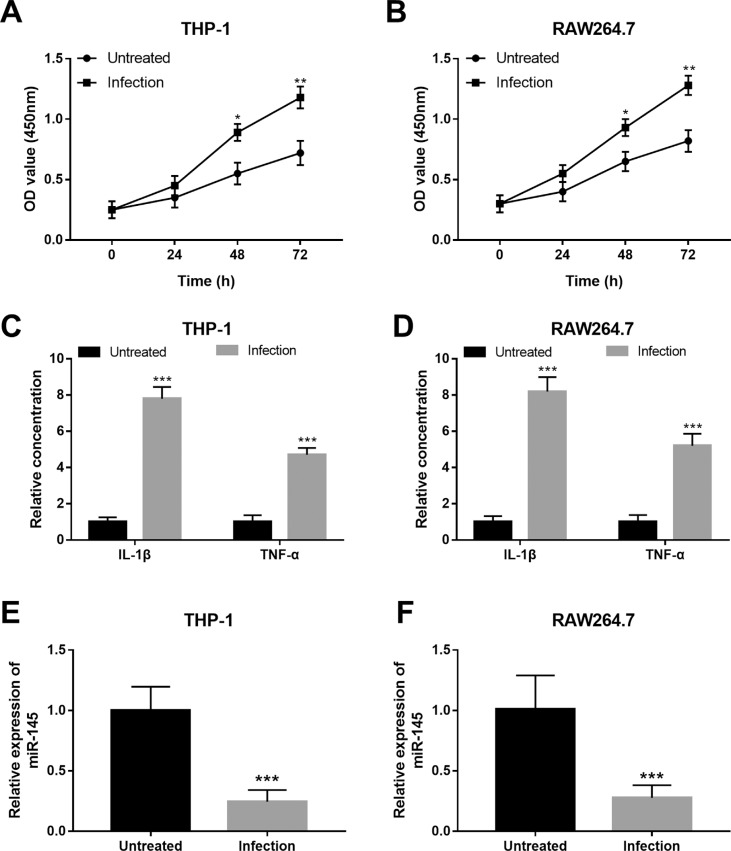
Effects of Mtb infection on cell viability, inflammatory responses and miR-145 expression in human macrophage THP-1 and murine macrophage RAW264.7 cells. A and B. The infected macrophage cell viability was promoted compared with the untreated cells. C and D. The inflammatory reaction was activated in the macrophages by Mtb infection. E and F. Expression of miR-145 was suppressed in the macrophages after the infection with Mtb. *P < 0.05, **P < 0.01, ***P < 0.001 vs. Untreated.

Two macrophage cell lines, THP-1 and RAW264.7, were used in the present study. After the infection of Mtb H37Rv infection, we observed that the infected macrophages exhibited the increased cell viability compared with the untreated cells (all *P* < 0.05, Figure 3A and 3B). In addition, the inflammatory cytokines IL-1β and TNF-α were both promoted following Mtb infection in the macrophages (all *P* < 0.001, Figure 3C and 3D). All these data revealed the promoted cell viability of infected macrophages and the infection-induced activation of the inflammatory response. Furthermore, the changes in the expression of miR-145 induced by Mtb infection were examined. As shown in Figure 3E and 3F, the expression of miR-145 was obviously downregulated by the Mtb infection in both the two macrophages compared with the untreated cells (all *P* < 0.001).

### Effects of miR-145 on cell viability and inflammation in macrophages upon Mtb infection

**Figure 4:**
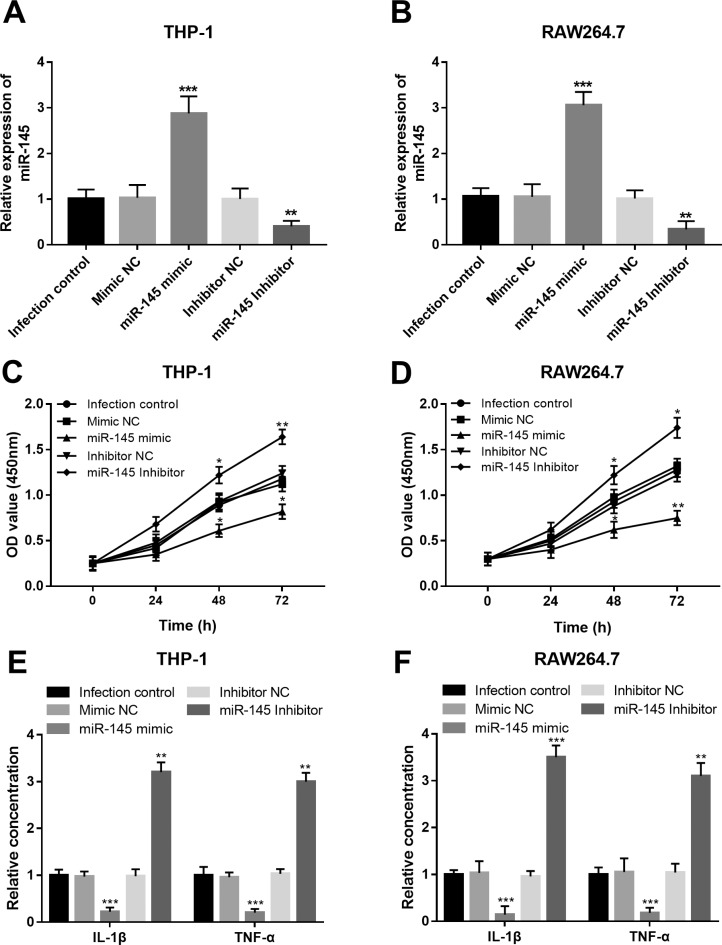
Effects of miR-145 on the cell viability and inflammation in the macrophages upon Mtb infection. A and B. Expression of miR-145 was successfully regulated in both THP-1 and RAW264.7 cells. C and D. The overexpression of miR-145 could suppress, whereas the downregulation of miR-145 could promote the cell viability of the macrophages. E and F. The concentrations of IL-1β and TNF-α in the macrophages upon Mtb infection were inhibited by the upregulation of miR-145, but were enhanced by the knockdown of miR-145. *P < 0.05, **P < 0.01, ***P < 0.001 vs. Infection control.

To uncover the functional role of miR-145 in the development of TB, this study performed in vitro experiments to investigate the effects of miR-145 on macrophage cell viability and Mtb infection-induced inflammation. As shown in Figure 4A and 4B, the expression of miR-145 in both the THP-1 and RAW264.7 cells was upregulated by the miR-145 mimic, and was downregulated by the miR-145 inhibitor (all *P* < 0.01). By the *in vitro* manipulation of miR-145, we observed that the cell viability of the infected macrophages was suppressed by the overexpression of miR-145, but was promoted by the knockdown of miR-145 (all *P* < 0.05, Figure 4C and 4D).

We examined the inflammatory response in the infected macrophages, and we found that the levels of IL-1β and TNF-α were all suppressed by the upregulation of miR-145 but were increased by the downregulation of miR-145 (all *P* < 0.01, Figure 4E and 4F).

### miR-145 directly regulates CXCL16 in macrophages

**Figure 5:**
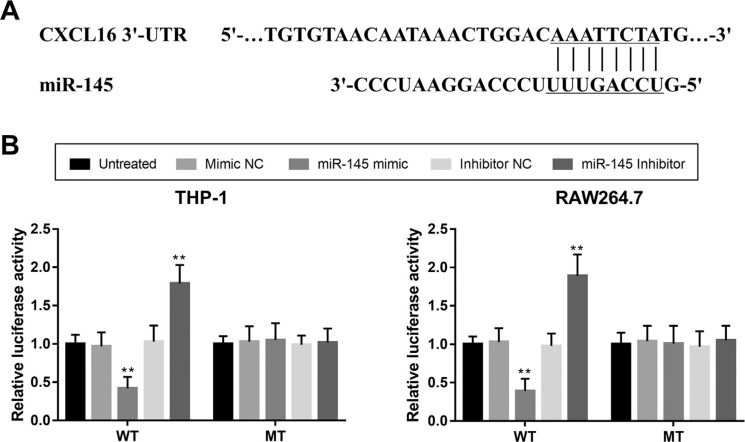
CXCL16 is a direct target gene of miR-145. A. A complete sequence of miR-145 was found in the 3’-UTR of CXCL16. B. Luciferase activity in WT group was suppressed by the overexpression of miR-145, but was promoted by the knockdown of miR-145 in both THP-1 and RAW264.7 cell lines. WT, wild type; MT, mutant type; **P < 0.01 vs. Untreated.

By the bioinformatics prediction, the 3’-UTR of CXCL16 has the complementary sequence of miR-145 (Figure 5A). Thus, luciferase reporter assay was performed in the two macrophages. As shown in Figure 5B, the luciferase activity in the WT group was significantly suppressed by the overexpression of miR-145 and promoted by the downregulation of miR-145 (all *P* < 0.05), whereas no change in the luciferase activity was found in the MT group in both the THP-1 and RAW264.7 cell lines.

## Discussion

TB induced by the infection with Mtb remains a global health burden. Macrophages are considered the first barrier against Mtb infection in TB patients. This study focused on the serum expression levels of miR-145 and its diagnostic value in the patients with TB, and further uncover the biological function of miR-145 by using the macrophages infected with Mtb. The present study found that the expression of miR-145 was significantly downregulated in TB patients, and the deregulated serum miR-145 expression was negatively correlated with the patients’ serum levels of IL-1β and TNF-α. By constructing ROC curves, we demonstrated the relative highly diagnostic value of miR-145 in distinguishing TB patients from the healthy controls. Moreover, the decreased miR-145 expression could also be used for the differentiation between ATB and LTB with considerable diagnostic accuracy. Furthermore, the macrophages showed enhanced cell viability and inflammatory responses after the infection with Mtb, and the expression of miR-145 was obviously inhibited by the Mtb infection. After *in vitro* regulation of miR-145, the increased cell viability and inflammatory responses induced by Mtb infection were all suppressed in the macrophages.

Deregulation of miRNAs has been highlighted in various human diseases (Ratnadiwakara *et al.*, 2018). The clinical significance of the abnormal miRNAs expression has attracted increasing attention in the diagnosis and prognosis of diseases, including TB (Puik *et al.*, 2017; Li and Zhang, 2019). For example, the expression levels of miR-146a and miR-146b were higher in papillary thyroid carcinoma, and served as a candidate diagnostic and prognostic biomarker for the patients with this malignancy (Qiu *et al.*, 2017). The increased serum expression of miR-411 in non-small cell lung cancer patients had high diagnostic accuracy in distinguishing cancer patients from healthy individuals (Wang *et al.*, 2017). The upregulated serum expression levels of miR-133 in the patients with lymphoma-associated hemophagocytic syndromes were determined as a useful biomarker for the diagnosis of this disease (Li *et al.*, 2017). For the diagnosis of subclinical atherosclerosis, Huang *et al.* (2017) found that the increased expression of miR-29b could be used as a useful biomarker (Huang *et al.*, 2017). In the patients with TB, the aberrant expression of miR-29a-3p in the plasma samples was demonstrated to perform a good distinguishing potential in discriminating ATB patients from healthy controls (Ndzi *et al.*, 2019). Another study by Wagh *et al.* (2017) provided evidence for the elevated miR-16 and decreased miR-155 to serve as two diagnostic biomarkers for the patients with TB (Wagh *et al.*, 2017). Therefore, identifying novel miRNAs that have distinguishing abilities for screening patients is urgently needed for the diagnosis of TB.

This study found a significant decrease in the serum expression levels of miR-145 in patients with TB compared with healthy controls. More importantly, lower serum miR-145 expression was observed in the ATB cases than in LTB patients. Moreover, the increased inflammatory responses in the TB patients, which were evidenced by the elevated IL-1β and TNF-α, were found to be negatively correlated with the serum expression levels of miR-145. Thus, we considered that miR-145 might be involved in the development of TB, especially the inflammatory processes. The further ROC analysis results suggested that the decreased miR-145 expression had good diagnostic performance to distinguish LTB and ATB patients form the healthy individuals. Notably, a considerable diagnostic value of miR-145 was also proved for the discriminating ATB patients from LTB patients. All these data revealed that the dysregulation of miR-145 might serve as an efficient diagnostic biomarker in TB patients. According to the previously published literatures, the diagnostic significance of miR-145 has been reported in acute myocardial infarction (Xue *et al.*, 2019) and ovarian carcinoma (Kim *et al.*, 2019). Our results provided a novel insight on the clinical value of miR-145 in TB.

It is known that the immune defence from macrophages is the first barrier against Mtb infection (Lou *et al.*, 2017). Thus, the interaction of Mtb and macrophages constitutes a pivotal step of TB development. In the present study, two types of macrophage were applied and shown increased cell viability and inflammatory reaction after the infection of Mtb. In a study by Furci *et al.* (2013) miR-145 was demonstrated to be downregulated in human macrophages upon Mtb infection (Furci *et al.*, 2013). Our study also obtained the decreased expression of miR-145 in the macrophages after Mtb infection, which was consistent with the previously reported result. To further understand the functional role of miR-145, the cell proliferation and inflammation were assessed in the macrophages with regulated miR-145. Compared with the infected control cells, Mtb-infected macrophages overexpressing miR-145 had inhibited cell viability and inflammatory responses, while the knockdown of miR-145 led to the opposite results, which suggested that miR-145 might act as a suppressor role during the development of TB by inhibiting the cell viability of infected macrophages and decreasing the infection-induced inflammatory reaction.

Previous mechanistic research has shown that miR-145 could regulate cell proliferation by targeting TGF-β1 in breast cancer (Ding *et al.*, 2017), by targeting NRAS in condyloma acuminatum (Liu *et al.*, 2018), and by regulating ADAM19 in retinoblastoma (Sun *et al.*, 2015). Some of these reported target genes of miR-145 have been demonstrated to serve pivotal roles in the progression of TB (Warsinske *et al.*, 2017). CXCL16, as a ligand of CXCR6, has been reported to be involved in the management of Mtb infection (Ashhurst *et al.*, 2019) and to mediate the inflammatory response (Jozefowski *et al.*, 2011). More importantly, CXCL16 has been determined as a mediator of the inhibiting effects of miR-145 on proliferation and inflammation in rat mesangial cells (Wu *et al.*, 2018). Thus, we wondered whether miR-145 suppress the proliferation and inflammatory reaction in macrophages also through targeting CXCL16. According to bioinformatics prediction and luciferase activity assay, we found that CXCL16 was a direct target gene of miR-145 in macrophages, indicating that the biological function of miR-145 in the progression of TB might be attained by targeting CXCL16. Although this study provides a novel insight into the clinical significance and biological function of miR-145 in TB, as well as the potential target underlying the molecular mechanisms, more investigations are necessary to confirm the role and mechanism of miR-145 acting in the development of TB.

In conclusion, all the data of this study revealed that the serum expression levels of miR-145 are decreased in the patients with TB and negatively correlated with the serum levels of inflammatory cytokines. The aberrant expression of miR-145 had good diagnostic performance for distinguishing TB patients from healthy controls and ATB cases from LTB cases. In the macrophages upon Mtb infection, the overexpression of miR-145 could suppress the cell viability of infected cells and the infection-induced inflammation. Thus, we believe that miR-145 may serve as an efficient diagnostic biomarker of TB, and the methods to upregulate miR-145 may become the novel therapeutic approaches for the treatment of TB.
